# A mitochondria-targeting artemisinin derivative with sharply increased antitumor but depressed anti-yeast and anti-malaria activities

**DOI:** 10.1038/srep45665

**Published:** 2017-04-03

**Authors:** Chen Sun, Yu Cao, Pan Zhu, Bing Zhou

**Affiliations:** 1 State Key Laboratory of Membrane Biology, School of Life Sciences, Tsinghua University, Beijing 100084, China

## Abstract

The potent anti-malarial drug artemisinins are additionally anti-tumorigenic and inhibitory to yeast growth. The action mechanism of artemisinins, however, is not well understood. Heme and mitochondrial membrane are both suggested to be involved in the action of artemisinins. Because heme is also synthesized in the mitochondrion, mitochondria appear to be a critical organelle for artemisinins’ activities. In this study, we synthesized a mitochondria-targeting artemisinin derivative by conjugating triphenylphosphonium (TPP) to artelinic acid (ARTa). ARTa-TPP displays far more potent anti-tumorigenic activity than its parent compound. In contrast, ARTa-TPP is much less active against yeast respiration growth and malarial parasites. Notably, ARTa-TPP is also associated with increased toxicity to other kinds of control mammalian cells. These results suggest divergent action modes for artemisinins against cancer cells and malaria or yeast cells. We conclude that mitochondrial targeting could substantially elevate the anticancer potency of artemisinins, but the accompanied increased toxicity to normal cells raises an alert. The mechanism regarding the opposing effects of TPP conjugation to ARTa on its anticancer and anti-malarial/anti-yeast potencies is discussed based on our current understandings of artemisinins’ action.

Artemisinin was originally isolated from the Chinese medicinal plant, *Artemisia annua L,* for its potent antimalarial activity^1^,^2^,^3^. A set of the first generation derivatives, all modified at the C10 position of artemisinin, was soon discovered by medicinal chemists to possess far superior activity or bio-availability^4^,^5^,^6^,^7^,^8^. Some of these class of drugs, altogether called artemisinins, have become the first line antimalarial medicine due to the development of malarial resistance to other antimalarial drugs^9^,^10^,^11^.

Apart from the antimalarial property, artemisinins have also been found, albeit less remarkably, to be associated with many other activities including inhibition of some other parasites, many cancer cell lines, and yeast *Saccharomyces cerevisiae*. The molecular bases underlying these various types of actions are, however, largely not clear^12^,^13^,^14^. In cancer cells lines, it has been shown that modulation of heme levels dramatically affects the anticancer properties of dihydroartemisinin(DHA) and artesunate^15^,^16^. Since direct reactivity of heme with artemisinins is well known in chemistry, it is natural to propose that a heme-mediated action activates artemisinins here, inducing a toxic effect through more or less promiscuous ROS damaging^17^,^18^,^19^,^20^. Recent proteomic studies confirmed that this action is indeed directed to a number of protein targets^21^,^22^. A key question, which is still hotly debated, is whether the antimalarial activity of artemisinin mimics its anticancer activity. In other words, whether a universal mode of action underlies the anticancer, antimalarial and possibly other activities of artemisinins. According to this “universal model”, one can argue that intracellular heme levels or available heme levels, together with variable cellular tolerances to free radicals, determine the differential toxicities of artemisinins against various kinds of cells.

We have reported that artemisinins additionally inhibit the growth of *S. cerevisiae* (hereafter “the yeast”) on non-fermentable media (mitochondrial-respiration dependent media)^23^,^24^. Later studies demonstrated two major types of activities of artemisinins in the yeast^25^,^26^. One involves depolarization of mitochondrial membrane potential, and the other a heme-mediated cytotoxicity. These two types of activities are distinctly separable. In fact, down-regulation of heme dramatically mitigates the heme-mediated cytotoxicity of DHA, but significantly increases the anti-mitochondrial potency of DHA and artemisinins. We proposed that the relatively specific anti-mitochondrial action and the less specific or general heme-mediated action of artemsinins may be competitive so that inhibition of the heme-mediated reaction makes more artemisinins available to act against the mitochondrial membrane^26^. For some mysterious reason hitherto unknown, mammalian mitochondrial membrane does not respond to artemisinins whereas those of malarial parasites and the yeast are highly sensitive^24^.

Final steps of heme synthesis occur in mitochondria, therefore mitochondria appear to be a key player in the action of artemisinins, either in the heme-mediated action mode or the heme-antagonized, mitochondrial membrane-depolarization mode. We asked then what will happen if we target artemisinins to mitochondria. In this work, we conjugated an artemisinin derivative, artelinic acid(ARTa), to mitochondria-targeting triphenylphosphonium(TPP) cation, to analyze the biological effects after this alteration. Interestingly, this modification greatly changed the biological property of the parental compound. Compared to ARTa, ARTa-TPP is many folds higher in anticancer potency but significantly diminished in its anti-yeast and antimalarial activity. These observations may have profound implications in the action mechanism of artemisinins and are thoroughly discussed.

## Methods

### Cell culture

HepG2, LoVo, PANC-1 and MCF-7 cancer cells were cultured in DMEM(Dulbecco’s Modified Eagle Medium) supplemented with 10% FBS, penicillin(100 U/ml) and streptomycin(100 ug/ml) at 37 °C in a humidified atmosphere of 5% CO_2_. Normal mammary epithelial cells MCF-10A were grown in DMEM/F12 supplemented with 5% horse serum, human epidermal growth factor, insulin cholera toxin and hydrocortisone. Human liver LO2 cells were grown in DMEM/F12 medium with 10% FBS. Primary kidney cells were obtained from 3-d-old mice. Whole kidneys were minced and cells were dissociated in 0.25% trypsin-EDTA in PBS buffer. The cells were centrifuged at 500 g for 10 min, and the pellet was resuspended in DMEM supplemented with 10% FBS. Kidney cells were tested in their first culture passage for the cytotoxic assay.

### Cytotoxic assay

Cells were inoculated into 96-well plates in 100 μL at plating densities depending on the doubling time of individual cell line. After incubating for 24 h, growing cells were treated with freshly prepared compounds diluted in media(100 μL) to obtain a final concentration ranging from 0 to 50 μM. Following 48 h compound exposure, 3-(4,5-dimethylthiazol-2-yl)-2,5-diphenyltetrazolium bromide (MTT) was added to the cells at a final concentration of 0.35 mg/ml. The culture medium was removed after 4 h of additional incubation, the formazan crystals were dissolved by addition of 150 μL DMSO and the absorbance at 570 nm was measured using a multi-well plate reader (Multiscan GO, Thermo). Cellular ATP levels were measured by the Cell Titer-Glo Luminescent Cell Viability Assay (Promega) using the plate reader(EnSpire^®^ Multimode Plate Reader, PerkinElmer). Data represent the percentage of live cells compared with the control group.

### *In vitro* antimalarial assay

*P. falciparum*(strain 3D7) was maintained in continuous culture. Briefly, the parasites were kept at 37 °C in human erythrocytes with complete medium (RPMI 1640 supplemented with Albumax II). Prior to assay initiation, the level of parasitemia of aliquot of a stock culture was measured by light microscopy following Giemsa staining. Compounds were added to cultures with 2% parasitemia and 2% hematocrit cultures in a total assay volume of 200 μL in 96-well plates, and incubated at 37 °C in a humidified air incubator containing 5% CO_2_. After 48 h of growth, 50 μL of SYBR Green I diluted in lysis buffer (10 mM Tris-HCl, 1 mM EDTA, pH 7.5 and 2% Triton X-100) was added to each well, and the contents were mixed until no visible erythrocyte sediment remained. After 30 min of incubation in the dark at 37 °C, fluorescence was measured by a microplate fluorometer (Fluoroskan Ascent, Thermo) with excitation and emission wavelengths centered at 485 and 538 nm, respectively.

### Yeast strains and growth inhibition analysis

Standard yeast media and growth conditions were used. *S. cerevisiae* BY4742 (*MATα his3*Δ*1 leu2*Δ*0 lys2*Δ*0 ura3*Δ*0*) was used in this study. Yeast was normally plated on YPD (2% glucose as carbon source) or YPGE (2% glycerol plus 2% ethanol as carbon source) agar plates. For growth testing on agar plates, yeast previously grown on YPD plates was 10-fold serial diluted with sterile ddH_2_O, and then spotted on YPD or YPGE plates with or without drugs (Spotting assay).

## Results

### ARTa-TPP significantly increased cytotoxic activity against cancer cells

**Figure 1:**
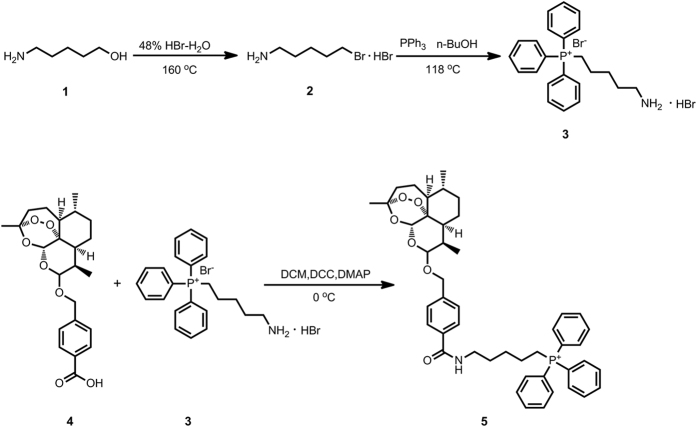
Synthesis of the mitochondria-targeting ARTa-TPP. **1**. 5-aminopentan-1-ol; **2**. 5-bromopentan-1-amine hydrobromide; **3**. triphenylphosphonium bromide; **4**. Artelinic acid (ARTa); **5**. ARTa-TPP.

Inspired by the current hypotheses for the mechanism of action of artemisinin, we designed and synthesized an artemisinin derivative, artelinic acid-TPP (ARTa-TPP), to explore how mitochondria -targeting might affect artemisinins’ activity. TPP is a lipophilic molecule that can easily permeate membranes, and due to its cationic nature, it selectively accumulates in mitochondria in a membrane potential dependent manner. As shown in Fig. 1, ARTa was conjugated with TPP via a pentyl spacer to form ARTa-TPP. Our choice of a 5-carbon alkyl spacer is because of our experience that a 2-carbon linker is not stable, and a pentyl linker has been successfully employed by others in a number of known TPP-derived conjugate (for references, see: refs 27, 28, 29). The structure of the derived ARTa-TPP was characterized by mass and NMR spectra (Supporting information).

**Figure 2:**
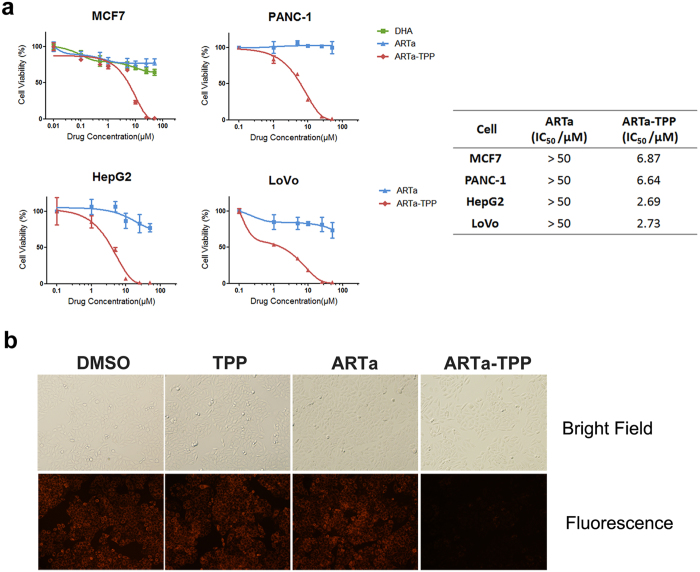
TPP fusion drastically increased the inhibitory potency of ARTa on cancer cells. **(a)** Cytotoxicity of ARTa and ARTa-TPP to human hepatoma cell line HepG2, human colon adenocarcinoma cell line LoVo, human pancreatic carcinoma epithelial-like cell line PANC-1 and human breast adenocarcinoma cell line MCF7. Cells were exposed to various concentrations of the compounds for 48 h followed by MTT assay. All assays were done in triplicate. **(b)** Microscope images of HepG2 cells upon incubation with TPP (25 μM), ARTa (25 μM), ARTa-TPP (25 μM) or with DMSO as control for 3 h. Tetramethylrhodamine ethyl ester (TMRE) was used as the mitochondrion probe. TMRE is very emissive when mitochondrial membrane potential is high.

The cytotoxic effect of ARTa-TPP was evaluated with a panel of cancer cell lines: HepG2, LoVo, PANC-1 and MCF-7 cells. Compared to ARTa, ARTa-TPP possessed significantly increased cytotoxicity against all of the tested cancer cells. As shown in Fig. 2a, after 48 h incubation with drugs, ARTa-TPP was highly cytotoxic toward these cancer cells in a dose-dependent manner, with IC_50_ values below 10 μM. In contrast, the parent compound ARTa had much milder effects on viabilities of the cells(IC_50_ values > 50 μM for all the tested cells, and even over 100 μM for some lines). These results indicated that conjugating a mitochondria targeting moiety TPP to ARTa dramatically enhances its activity against cancer cell lines.

To confirm that ARTa-TPP indeed wreaks havoc on the mitochondria of the cancer cells, we used tetramethylrhodamine ethyl ester (TMRE), which is sensitive to mitochondrial membrane potential, to analyze a possible membrane potential loss in the HepG2 cells. The fluorescence signal was much weaker after ARTa-TPP treatment, indicating a damaged mitochondrion (Fig. 2b). This data is consistent with a very recent report showing a similar fusion molecule of artemisinin also disrupted mitochondrial functions, accompanied with much increased *in vitro* anticancer activities^30^.

### Mitochondrial targeting decreased the *in vitro* anti-malarial activity of ARTa

**Figure 3:**
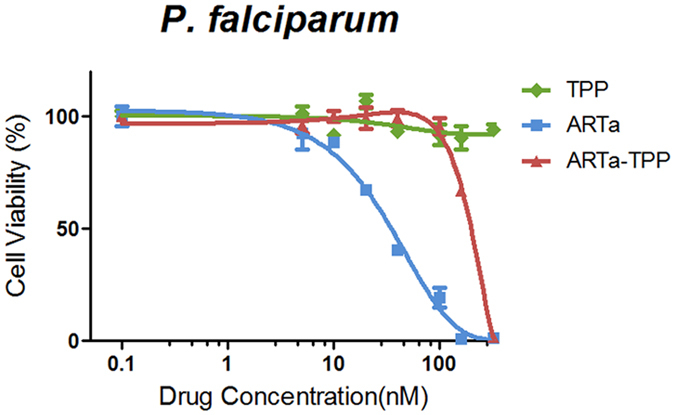
Inhibitory effects of malaria parasites by ARTa and ARTa-Tpp. Parasites were cultured for 48 h in the presence of various concentrations of drugs. Parasite viability was measured by fluorometric detection after SYBR Green I staining. TPP tagging to ARTa reduced its antimalarial efficacy.

Our previous study indicated that mitochondria are an important direct target in the antimalarial action of artemisinins^23^,^24^. The action appears to be mediated by depolarization of the mitochondrial membrane. We hypothesized that ARTa accumulation in mitochondria by attaching TPP might enhance its antimalarial activity. In order to test this possibility, ARTa-TPP was analyzed for its antimalarial activity along with ARTa against the 3D7 strain of *P. falciparum*. As shown in Fig. 3, ARTa was, as expected, very active against the parasite with an IC50 of 44 nM. Surprisingly, ARTa-TPP showed a dramatic loss of antimalarial activity; ARTa-TPP conferred a negligible impact on the viability of parasites at 160 nM, but exerted a strong inhibition at 320 nM. Since it is known that hydrophobicity of artemisinins or their metabolites play a vital role in the antimalarial actions, we suspect this drop of antimalarial activity may be caused by the reduced hydrophobicity of ARTa-TPP due to the cationic nature of TPP moiety (Discussion).

### Mitochondria-targeting of ARTa increased yeast-inhibitory activity on fermentable media but not on respiration-dependent media

**Figure 4:**
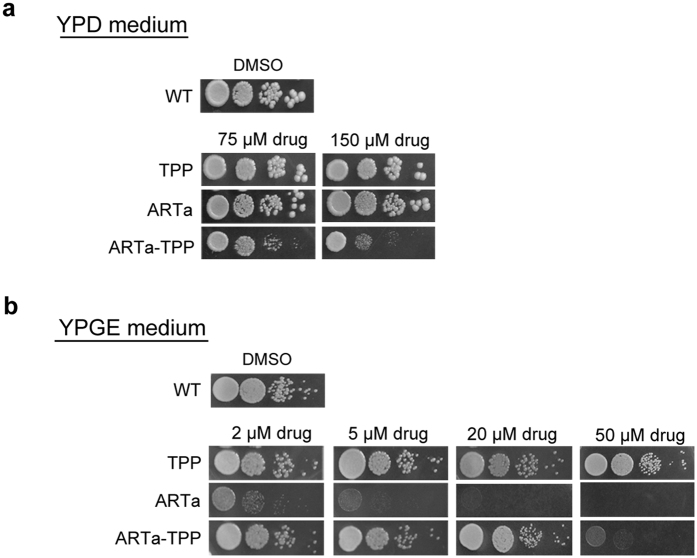
In yeast, inhibitory activity of ARTa-TPP is increased against fermentable growth but decreased against respiratory growth. **(a**) Yeast inhibition of ARTa and ARTa-TPP on YPD plates. Yeast was spotted on agar plates with or without drugs by 10-fold serial dilutions in sterile water. ARTa was very inefficient in inhibiting yeast on fermentable YPD media. ARTa-TPP inhibited yeast growth better than the parent drugs. **(b)** Spotting assay of artemisinins on non-fermentable media(YPGE). Very low concentrations of ARTa could inhibit yeast respiratory growth. In contrast to the effect on fermentable growth on YPD, TPP conjugation greatly reduced the efficacy of ARTa on non-fermentable media. TPP and DMSO (the solvent) were also tested and used as the control.

Baker’s yeast *S. cerevisiae* can grow in respiration-dependent (energy generation through mitochondrial membrane potential as a result of respiration) or -independent (energy generation through fermentation) manner. It has been shown that the respiratory growth (i.e., on non-fermentable media) of yeast is especially sensitive to artemisinins^23^,^24^. On fermentable media, yeast is much less sensitive to artemisinins^26^. The former inhibition is attributed to a partial mitochondrial membrane depolarization and the latter a relatively general, and mostly heme-mediated toxicity^26^. We asked what effects of the mitochondria-targeting will have on these activities. Yeast growth was severely inhibited on non-fermentable media (requiring functional mitochondrial respiration) YPGE by ARTa (Fig. 4b); at 5 μM the growth of yeast was nearly completely arrested whereas no significant growth inhibition was detected on fermentable media(growth not requiring functional mitochondria) YPD even at a concentration of ARTa as high as 150 μM. Meanwhile, the toxicity of ARTa-TPP was more pronounced when yeast cells were grown on YPD media (Fig. 4a). However, a sharp decrease of activity was observed for ARTa-TPP on YPGE media, suggesting a reduced efficacy in effecting mitochondrial membrane depolarization. Notably, these disparate inhibitory activities of ARTa and ARTa-TPP on yeast grown on YPD and YPGE correlated well with their divergent activities against cancer cells and malarial parasites.

### ARTa-TPP acts in a general mechanism and is increased in cytotoxicity even against normal cells

The above observation that ARTa-TPP is associated with a significantly exacerbated anticancer activity but much reduced potency against malarial parasites and yeast growth on non-fermentable media, suggested to us that ARTa-TPP, despite mitochondria-targeted, is less effective conferring mitochondrial membrane depolarization to yeast and malarial parasites. Although the specific mitochondrial membrane-depolarizing action of artemisinins is not understood mechanistically, in yeast it is found to be antagonized, instead of potentiated, by heme^25^,^26^. We reasoned that the increase of inhibition activity of ARTa-TPP against cancer cells might be due to its potentiation by heme, just as the inhibition of artemisinins against fermentable (non-respiratory) growth of yeast^26^, and dihydroartemisinin (DHA)-induced cytotoxicity in cancer cells^15^.

**Figure 5:**
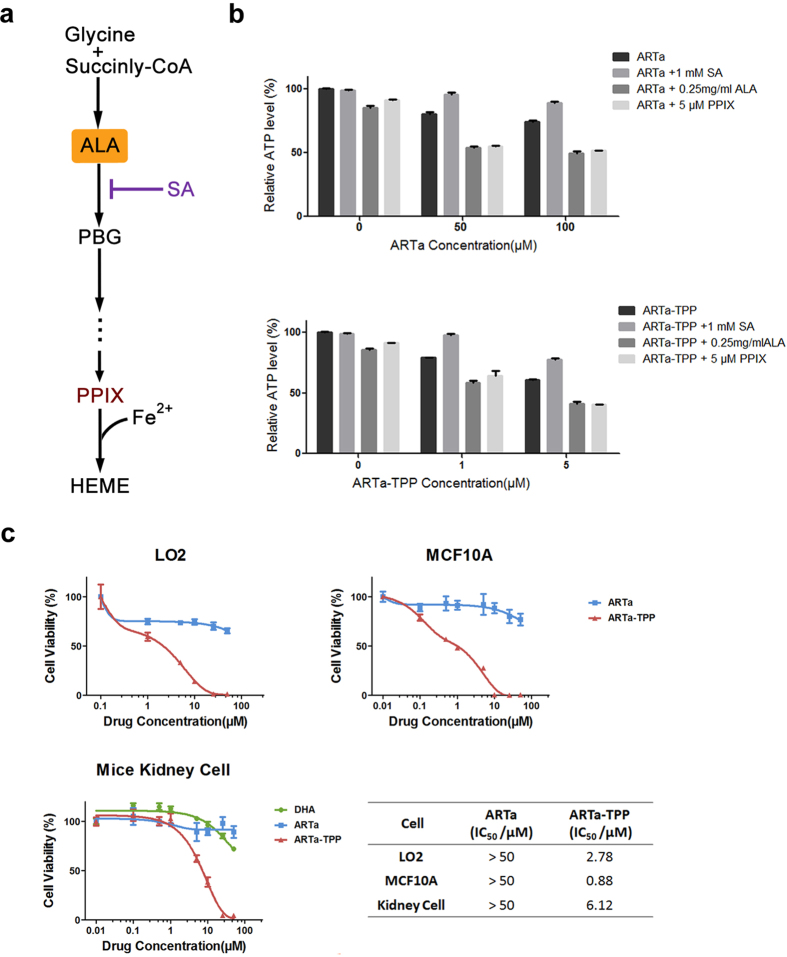
ARTa-TPP could also inhibit normal mammalian cells. (**a**) A schematic representation of the heme biosynthetic pathway in cells. SA, succinylactone; ALA, δ-aminolevulinic acid; PPIX, protoporphyrin IX; PBG, porphobilinogen. **(b**) Cell viability of HepG2 cells cultured with ARTa, ARTa-TPP with SA, ALA or PPIX, measured by ATP level changes. Cells were pre-incubated with or without SA to inhibit heme synthesis or ALA and PPIX to enhance the heme synthesis prior to addition of artemisinin drugs. ALA and PPIX further increased the cytotoxicity of ARTa and ARTa-TPP, while SA could restore the growth of the cells. Note much higher concentrations of ARTa were used than ARTa-TPP. (**c**) Cytotoxicity of ARTa and ARTa-TPP to normal mammalian cells lines including human mammary epithelial cells MCF-10A, liver cells LO2 and primary mice kidney cells. Cells were exposed to various concentrations of the compounds for 48 h followed by MTT assay. All assays were done in triplicate.

To investigate whether heme might participate in the cytotoxic effects of ARTa-TPP, we modulated the intracellular heme levels by supplementing the heme synthesis inhibitor succinylactone(SA), the substrate δ-aminolevulinic acid (ALA) and the precursor protoporphyrin IX(PPIX) into the culture media (Fig. 5a). ATP levels were measured as an indicator of cell viability. As shown in Fig. 5b, changing the heme level in cells could alter the anticancer effect of both ARTa and ARTa-TPP: both drugs could decrease the intracellular ATP levels at different concentrations, but co-incubation with SA could partially suppress this cytotoxic effect. In addition, co-incubation with 0.25 mg/ml ALA or 5 μM PPIX would enhance the cytotoxicity of ARTa and ARTa-TPP, resulting in further decrease of ATP levels relative to that of the control cells. These results suggest that the anticancer toxicity of ARTa-TPP is at least partially heme-mediated, i.e. heme plays a potentiating effect in the anticancer activity of ARTa-TPP.

Generously speaking, heme is a ubiquitous molecule despite that its intracellular levels may differ among different types of cells. Heme-induced toxicity therefore will happen more or less across many cell types. To confirm this assumption, we performed cell viability analysis in nonmalignant cells including mammary epithelial cells MCF-10A and liver cells LO2. As shown in Fig. 5c, ARTa (up to 100 μM) had very minor effect on viabilities of these cells. In contrast, significant cytotoxic effect was observed in ARTa-TPP treated cells, with much lower IC50 values compared with ARTa. A caveat is that these non-cancer cells, being cell lines, are not completely “normal” cells. We therefore additionally used a primary cell culture, derived directly from dispersed cells of baby mouse kidneys. Again, similar observation was made; ARTa-TPP was substantially more toxic than ARTa. These results indicate that ARTa-TPP is endowed with increased anti-cancer activity through a general mechanism existent in normal cells.

## Discussion

Due to potentially important roles of mitochondria in the action of artemisinins, we designed and synthesized a mitochondria-targeted artemisinin homologue, ARTa-TPP. ARTa-TPP exhibited sharply increased activity against cancer cell lines while much reduced anti-yeast and anti-malarial activity. The divergence in inhibition potency between cancer cells and malaria parasites/yeast suggests to us different mechanisms might be involved in these inhibitions. Notably, anti-yeast activity (on non-fermentable media, i.e., mitochondrial respiration-dependent media) and anti-malaria activity are correlated, consistent with a previous report showing that some other artemisinin derivatives which have been examined have also well-correlated anti-yeast and anti-malarial activities^24^. On the other side, many artemisinin-based compounds have been synthesized to search for better antitumor agents, only to find some superior anti-tumor agents with poor antimalarial activity, or vice versa^4^,^31^.

During the preparation of this manuscript, another work was published showing that a highly similar artemisinin and TPP fusion compound is also much more potent against cancer cell lines, consistent with our findings. Fusion of artemisinin to TPP indeed concentrated artemisinin to the mitochondria^30^. However, its activity against non-cancer cell lines was not documented or examined, nor was its activity against malaria parasites and yeast.

Mechanism of action of artemisinins is still a hotly debated issue and no clear answer is in sight. Our previous functional or genetic dissection of artemisinins’ action revealed two major types of biological properties for artemisinins^25^,^26^. One is a specific mitochondria-depolarizing function, and another less specific and much less potent heme-mediated cytotoxicity. The anti-mitochondrial action of artemisinins against yeast and malarial parasites is very clear and is directly established with isolated mitochondria from these cells^24^. The anti-mitochondrial action of artemisinins was supported by a later study using whole parasites showing that instant membrane depolarization, including mitochondrial membrane, was observed after artemisinins treatment^32^. This kind of depolarization occurs at low levels of artemisinins. For example, 100 nm and 1 μM artemisinin could respectively induce obvious and immediate depolarization in purified malarial and yeast mitochondria^24^. In addition, the membrane depolarization happened less than several minutes (likely in seconds) and presumably long before observable morphological changes of mitochondria^13^,^32^,^33^. These pieces of evidence strongly suggest that mitochondrial depolarization contributes at least partly to the inhibitory effects of artemisinins against malaria. Interestingly, mitochondrial depolarization after artemisinins treatment occurs only in some specific types of cells such as malarial parasites, the yeast *S. cerevisiae*, but not in mammalian cells^24^, indicating some unique features of mitochondria define this type of action. In this regard, there are some indirect pieces of evidence, though far from certain yet, implicating the involvement of electron transport chain (respiration chain) in this. Modulating respiration activity through regulation of NDH2 (alternative NADH dehydrogenase, including Ndi1 and Nde1 in yeast) activity in yeast could alter artemisinin sensitivity. Specifically, overexpressing NDH2 (Ndi1) increased yeast sensitivity to artemisinin whereas removal of Ndi1 reduced yeast sensitivity to artemisinin^23^. As a consequence, respiration chain was proposed to be likely involved in the activation of artemisinin, and the activated artemisinin then locally inflicts harms leading to membrane depolarization. Notably, this result was often misinterpreted and incorrectly cited in literatures as NDH2 being the target of artemisinins. Were NDH2 the target of artemisinins, it would have been expected that NDH2 overexpression would reduce the sensitivity and NDH2 reduction increase the artemisinin sensitivity, a result opposite to what have been observed. Consistently, low levels of artemisinins do not suppress respiration of yeast and malarial parasites^24^,^26^.

Although it is known that some of the yeast findings can be reproduced in malarial parasites (for example, the anti-mitochondrial action of artemisinins), it is not yet certain at this stage whether all or most of the yeast results hold true in malarial parasites. In yeast, both the heme- and mitochondria-mediated actions are observed, with the anti-mitochondrial activity a more potent action. In malaria parasites, a long held view is that heme plays a key part in artemsinin’s action. Heme is a ubiquitous molecule occurring in all types of cells. However, its levels in different cells are not uniform. Accordingly, heme-mediated toxicity of artemisinins potentially could happen, more or less, in all cells. In other words, the level of damage incurred by this fashion may depend on how much “available” heme exists, and how vulnerable the cells are to the inflicted damages. This type of heme-mediated artemisinins’ action, when observed in yeast grown on fermentable media, requires significantly higher levels of drugs compared to the anti-mitochondrial action^26^. Technically speaking, because fermentation enables yeast growth independent of the ATP formation driven by mitochondrial membrane potential, on fermentable media the anti-mitochondrial action of artemisinins, a partial membrane depolarization, is in fact “invisible”, making heme-mediated toxicity of artemisinins observable.

Several recent papers provided further pieces of evidence supporting that heme may work as an activator for artemisinins. For example, Tilley’s group has shown that with tightly synchronized parasites exposed to short drug pulses, ring-stage parasites can exhibit >100 fold lower sensitivity than trophozoites, consistent with the finding that ring stage parasites have much less hemoglobin digestion. Despite this, the very early ring-stage parasites are still super-sensitive to artemisinin^34^. Deletion of falcipain-2, an enzyme involved in hemoglobin degradation, significantly decreases Artemisinin sensitivity in the short pulse assays^34^,^35^. Also, Wang *et al*.^21^ have shown that heme is responsible for artemisinins’ activation to react and conjugate with many cellular proteins. Nevertheless, Khan’s group has reported that replication of Plasmodium in reticulocytes can occur without hemozoin formation, resulting in chloroquine resistance, but their sensitivity to artesunate, an artemisinin derivative, also thought to be dependent on hemoglobin degradation, is retained^36^.

**Figure 6:**
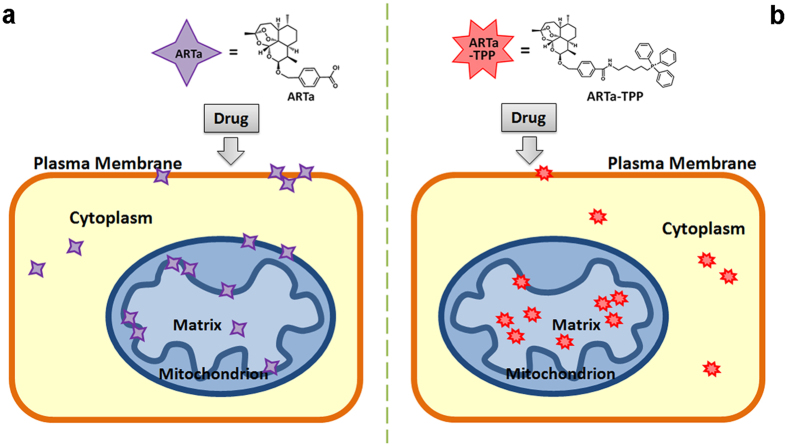
A schematic model to explain the divergent effects of TPP fusion to ARTa. (**a**) ARTa is a lipophilic molecule and preferably resides in the membrane system including the mitochondrial membrane, where it acts in an unclarified mechanism to partially depolarize the membrane of certain kinds of cells such as yeast and malarial parasites. This type of action is specific in the sense that it does not happen in all types of cells and the damage is local and limited, and at the beginning even likely reversible to some extent. (**b**) Lipophilicity of ARTa is altered after the ionic TPP fusion, making ARTa-TPP preferably reside in the matrix instead of the membrane. This redistribution makes it no longer an efficient membrane depolarizer. Instead, the matrix residence may inflict a general damage to the mitochondrion, causing an overall breakdown and a secondary membrane depolarization. This relatively general action happens in most, if not all, types of cells and requires higher dosages of the drugs.

What exactly happened to mitochondrial-targeting of ARTa so that it is so much more anti-mitochondrial in cancer cells but much less anti-mitochondrial in malarial parasites? In mammalian cells including cancer cells, normal artemisinins do not elicit serious harm to mitochondrial membrane depolarization even at high dosages, whereas yeast and malaria parasite mitochondrial membranes are highly sensitive to artemisinins^24^. The sensitive, albeit partial, mitochondrial depolarization action happened in yeast and malarial parasites is even reversible in short terms, meaning that washing after a short time artemisinin treatment can mostly reverse the membrane depolarization^24^. This suggests that this type of membrane depolarization occurred in yeast and malarial parasites, enabled by low amounts of artemisinin, is a partial/mild and specific action, but not a consequence from overall mitochondrial damage. On the other hand, TPP fusion of artemisinins will concentrate them in the mitochondria, causing a general type of damaging action against mitochondria, and as consequence, lead to a secondary membrane potential loss. One possibility might be that membrane residence of artemisinins is necessary to enable a highly efficient but partial membrane depolarization process. Since lipophilicity of artemisinins has been shown to be a key correlating factor in their antimalarial efficacy^37^, it is thus possible that the drop of activity of ARTa-TPP arises from the alteration of its lipophilic characteristics. When artemisinins are able to be distributed and enriched to the membrane they can efficiently cause dysfunction to the membrane, and when they do not reside there, their anti-membrane potency might be greatly compromised and the membrane potential loss observed is a secondary event and likely more extensive, happened at relatively higher dosages (Fig. 6).

Strictly speaking, before the anti-malarial action mechanism of artemisinins is finally certain, other possibilities cannot be excluded to explain the observed phenomenon in this study. In malarial parasites, in addition to de novo synthesis in the mitochondria, hemin is abundant in the malarial vacuole from degraded hemoglobin of red blood cells. Free heme is normally highly toxic to the cell, and the hemin in the vacuole is trapped and detoxified in the form of hemozin. It is not known how much of this form of hemin is available to the cell. As a result of this complexity and uncertainty, the loss of antimalarial activity of ARTa-TPP could be attributed to another cause, i.e., by targeting artemisinin to mitochondria less artemisinin might be available to the vacuole, where hemin in the form of hemozin is abundant. Along this line of thinking, vacuolar hemin should be active (becoming heme and available) and mediate the antimalarial activity of artemisinins in the vacuole. Nevertheless, in yeast, this unique style of hemin biology (accumulation of heme in the vacuole from hemoglobin breakdown) does not happen, so this is an unlikely scenario for the anti-yeast action of artemisinins. Intriguingly, among the several artemisinin derivatives we examined so far, including ARTa-TPP of this report, anti-yeast (on non-fermentable media) and anit-malarial activities are well correlated, pointing to the possibility of a shared mode of action between these two types of cells.

## Additional Information

**How to cite this article:** Sun, C. *et al*. A mitochondria-targeting artemisinin derivative with sharply increased antitumor but depressed anti-yeast and anti-malaria activities. *Sci. Rep.* **7**, 45665; doi: 10.1038/srep45665 (2017).

**Publisher's note:** Springer Nature remains neutral with regard to jurisdictional claims in published maps and institutional affiliations.

## Supplementary Material

Supplementary Information
