# Supplementary material for: A mitochondria-targeting artemisinin derivative with sharply increased antitumor but depressed anti-yeast and anti-malaria activities
Source: Sci Rep. 2017 Apr 3;7:45665. doi: 10.1038/srep45665 (PMC5377301; doi:10.1038/srep45665)
Supplement: Supplementary Information [file srep45665-s1.pdf]

A mitochondria-targeting artemisinin derivative with sharply increased antitumor but depressed anti-yeast and anti-malaria activities

Chen Sun[1], Yu Cao[1], Pan Zhu, Bing Zhou*

State Key Laboratory of Membrane Biology, School of Life Sciences, Tsinghua University, Beijing 100084, China

*Corresponding author. Tel: +86-62795322; Fax: +86-62772253; E-mail: zhoubing@mail.tsinghua.edu.cn.

1 C.S. and Y.C. contributed equally to this article.

Supplementary Information

Chemical synthesis

Artelinic acid-TPP was prepared as follows:

5-aminopentan-1-ol (3.0g, 29 mmol) was dissolved in a saturated HBr aqueous solution (34mL) and stirred in a sealed vessel at ambient temperature for 20 min. Subsequently, the vessel was transferred to a pre-heated oil bath (160°C) and kept stirring at the same temperature for 5h. The reaction mixture was cooled down to room temperature, and the solvent was evaporated. Acetone was added to dissolve the residue, followed by addition of ethyl acetate. The precipitation was collected and purified on chromatographic column with dichloromethane/methanol (20/1,v/v) to give a white solid(2 g，28%) .

A solution of the above obtained solid(0.7 g, 2.86 mmol) and triphenylphosphine(1.4 g, 5.3 mmol) in butyl alcohol (6mL)was refluxed at 120°Cfor 6h before cooling down to room temperature. To the reaction mixture were added benzene and ether, and the resulting precipitation was washed with ether until it was not sticky. The solid was then re-dissolved in ethanol, and ether was added. The precipitation was collected and purified on chromatographic column with dichloromethane /methanol(20/1,v/v) to give(5-aminopentyl) triphenylphosphonium bromide(0.8g，55%).

To a solution of artelinic acid(0.5 g, 1.2 mmol) in CH_2Cl_2(25 mL) under argon protection was sequentially added DCC(275 mg，1.32 mmol)，DMAP(50 mg，0.25 mmol) and(5-aminopentyl) triphenylphosphonium bromide(760 mg, 1.5 mmol).After stirring at 0°C for 6h,the reaction mixture

was filtrated and the filtrate was evaporated to give the crude product, which was purified on chromatographic column with dichloromethane/methanol(20/1,v/v) to give pure artelinic acid-TPP(300 mg, 33.5 %).^{1}H NMR($CDCl_3$,200 MHz) δ8.05(d, Ar, 2H), 7.78-7.62(m, Ph_3, 15H), 7.28(d, Ar, 2H), 5.40(s, artemisinin1H), 4.85(s, CH_2), 4.44(d, artemisinin.1H), 3.68-3.63(m, artemisinin.2H) , 3.44-3.42(m, artemisinin.2H), 2.60-0.81(m, artemisinin.6H). MS[ESI^{+}]m/z 748.4 [M^{+}].

^{1}H NMR and MS for TPP

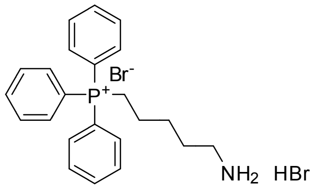

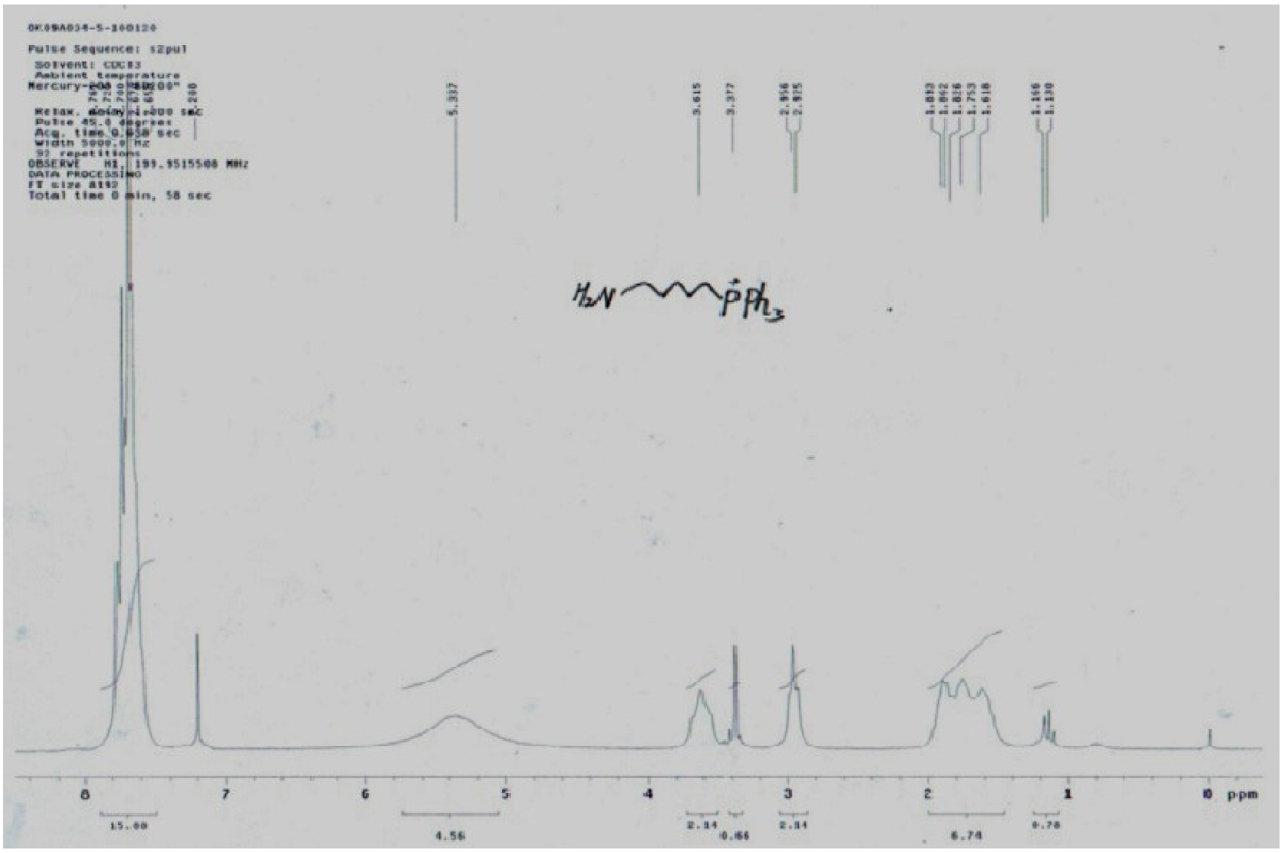

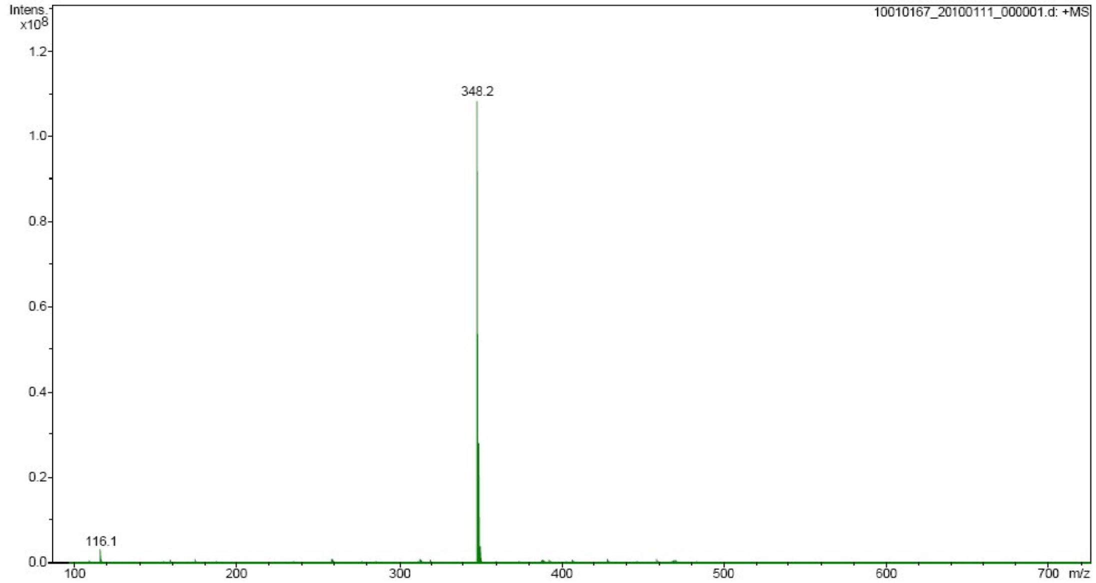

^{1}H NMR and MS for ARTa-TPP

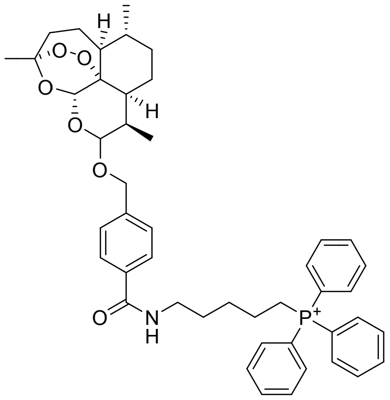

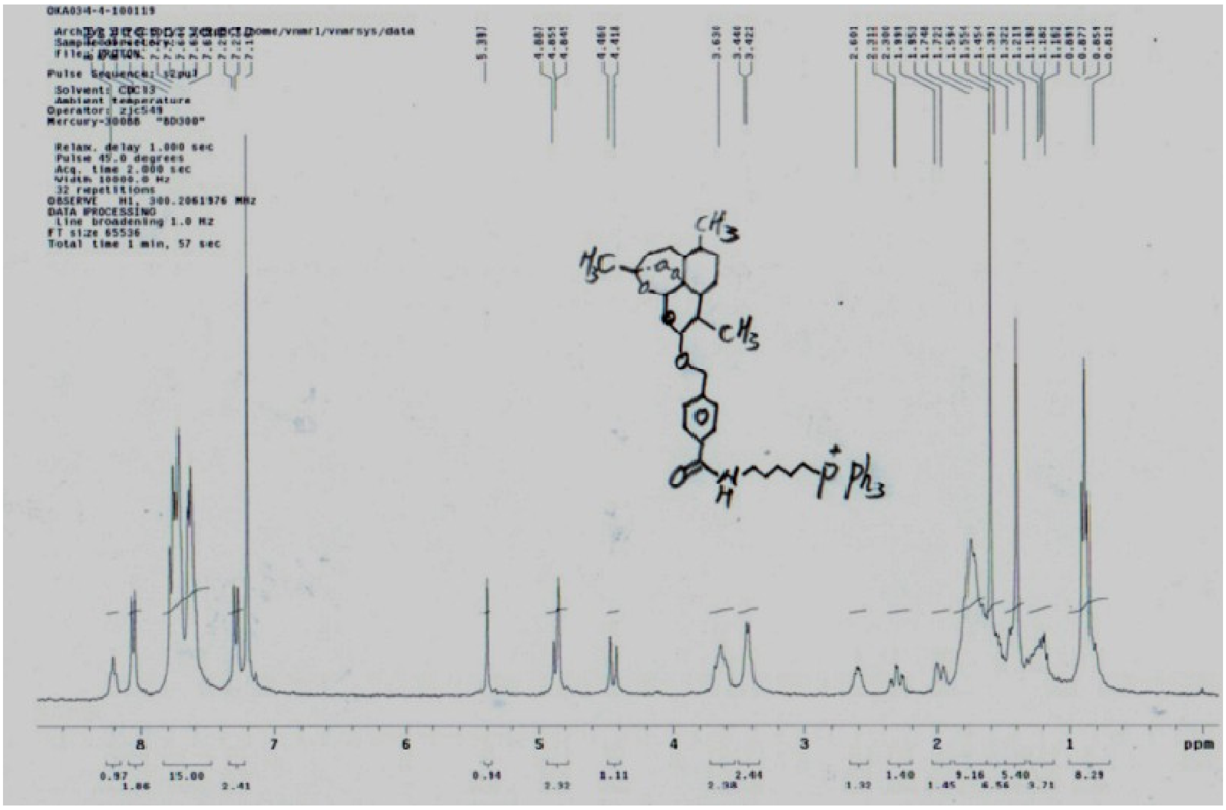

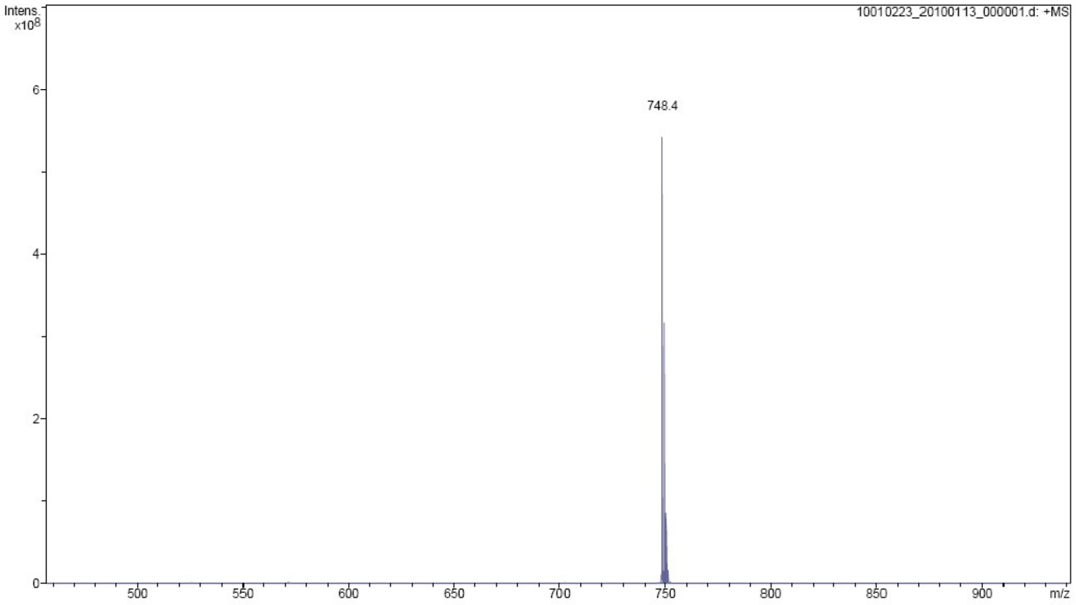
Intens.
x10^8
10010223_20100113_000001.d: +MS
748.4
6
4
2
0
500
550
600
650
700
750
800
850
900
m/z
